# DAMAC: A Delay-Aware MAC Protocol for Ad Hoc Underwater Acoustic Sensor Networks

**DOI:** 10.3390/s21155229

**Published:** 2021-08-02

**Authors:** Ahmed Al Guqhaiman, Oluwatobi Akanbi, Amer Aljaedi, Adel R. Alharbi, C. Edward Chow

**Affiliations:** 1 Department of Computer Science, University of Colorado at Colorado Springs, Colorado Springs, CO 80918, USA; oakanbi@uccs.edu (O.A.); cchow@uccs.edu (C.E.C.); 2 Department of Computer Networks and Communications, College of Computer Sciences and Information Technology, King Faisal University, Al-Ahsa 31982, Saudi Arabia; 3 College of Computing and Information Technology, University of Tabuk, Tabuk 71491, Saudi Arabia; aaljaedi@ut.edu.sa (A.A.); aalharbi@ut.edu.sa (A.R.A.)

**Keywords:** media access control protocols, quality of service, underwater acoustic sensor networks, propagation delay, concurrent transmissions, underwater nodes

## Abstract

In a channel shared by several nodes, the scheduling algorithm is a key factor to avoiding collisions in the random access-based approach. Commonly, scheduling algorithms can be used to enhance network performance to meet certain requirements. Therefore, in this paper we propose a Delay-Aware Media Access Control (DAMAC) protocol for monitoring time-sensitive applications over multi-hop in Underwater Acoustic Sensor Networks (UASNs), which relies on the random access-based approach where each node uses Carrier Sense Multiple Access/Collision Avoidance (CSMA/CA) to determine channel status, switches nodes on and off to conserve energy, and allows concurrent transmissions to improve the underwater communication in the UASNs. In addition, DAMAC does not require any handshaking packets prior to data transmission, which helps to improve network performance in several metrics. The proposed protocol considers the long propagation delay to allow concurrent transmissions, meaning nodes are scheduled to transmit their data packets concurrently to exploit the long propagation delay between underwater nodes. The simulation results show that DAMAC protocol outperforms Aloha, BroadcastMAC, RMAC, T${}_{u}$-MAC, and OPMAC protocols under varying network loads in terms of energy efficiency, communication overhead, and fairness of the network by up to 65%, 45%, and 726%, respectively.

## 1. Introduction

In recent years, UASNs have been considered to be the most reliable solution to support several underwater applications [1,2], which are classified into monitoring, disaster prevention, military, assisted navigation, and sports [3,4]. Each one of these categories can be further classified into different target applications where each application has different QoS requirements and hence requires different solutions [1,5]. Similarly, the network architecture of underwater nodes can be organized into the categories of one-dimensional (1-D), two-dimensional (2-D), three-dimensional (3-D), and four-dimensional (4-D); and, the QoS requirements of different underwater applications require different network architecture [6]. In supporting underwater applications, some MAC protocols waste network resources and need further attention to meet the QoS requirements of target applications [7,8]. The oil/gas industry is considered critical infrastructure to several countries as it helps to improve their economic competitiveness and growth [6,9]. In the last decade, many incidents have occurred, such as the Deepwater Horizon oil spill in the Gulf of Mexico, which resulted in 11 people killed, 3.19 million barrels of oil entering and damaging the Gulf ecosystem, and a cost in damages estimated by British Petroleum (BP) of about $62 billion [10,11,12]. This type of disaster can be avoided by implementing underwater nodes to monitor the status of the oil/gas pipeline. To efficiently monitor this type of application, underwater nodes must use the most appropriate MAC protocol approach in such a way that multiple underwater nodes can transmit critical data to the sink node while using low energy consumption per byte, low communication overhead, and ensuring that underwater nodes have equal opportunity to reserve the shared channel (fairness of network). Therefore, to support such a time-sensitive application, it is necessary to enhance the underwater communication relative to the Energy Efficiency (EE), Communication Overhead Ratio (COR) and Fairness Index (FI). Solutions that exist in Terrestrial Wireless Sensor Networks (TWSNs) for time-sensitive applications cannot be implemented in UASNs, due to acoustic channel characteristics, including limited bandwidth, high path loss, multipath effects, long and variable delay, high noise, and doppler spread [1,13,14,15,16,17]. Specifically, due to the characteristics of acoustic channel, using the handshake-based approach to avoid collision is considered expensive. Therefore, considering the long propagation delay to schedule data transmission can be a promising strategy [18,19,20,21], which can allow concurrent transmissions with no collision. To achieve this goal, it is necessary to consider the amount of data that must be transmitted to the sink.

The MAC protocol we propose here reduces collisions by considering the long propagation delay without negotiation between underwater sensors. This enhances the network performance in several metrics [18,19,20,21]. The proposed MAC protocol avoids the overhead required by the handshake-based approach by sensing the channel status and considering the long propagation delay of acoustic channel. This helps nodes to know when they can transmit, thus reducing the collision rate. To achieve this, scheduling algorithms are a critical factor to enhancing network performance [21,22]. Therefore, we propose a scheduling algorithm to resolve the collision problem and poor network performance. The schedule is typically a set of time slots in which we allow several nodes to transmit their packets concurrently while other nodes must be in idle state, thus reducing Energy Consumption (EC) [23]. Ultimately, this helps to reduce the amount of energy consumed relative to the number of bytes received and hence extends the network lifetime. The length of the time slot is adequate for data packets to reach the sink node plus allows for a short guard time. The guard time here is necessary to avoid the spatio-temporal uncertainty problem. The goal of this research was to develop the DAMAC to meet the requirements of time-sensitive applications by minimizing Total number of Collisions (TC), thus reducing delay [24,25]. Common network performance metrics, such as End-to-End Delay (E2ED), Packet Delivery Ratio (PDR), Throughput (Thpt), and EC are critical, and their improvement should be a strong focus of the field of UASN research. Therefore, it is necessary to develop a MAC protocol that can enhance these metrics to improve network performance in UASNs. To provide a complete performance analysis of a MAC protocol, it is also critical to measure the EE, COR, and FI. Table 1 summarizes some of the common issues encountered in the underwater environment and the requirements of UASNs.

The handshake-based approach is widely used to resolve collision issues, but it results in low channel use, low network throughput, and high E2ED due to the characteristics of acoustic channels [28,29,30,33]. Therefore, in this paper we propose DAMAC protocol, which avoids the handshaking packets prior to data transmission and schedules nodes to transmit their packets while reducing the collision rate [14,20,31,32]. Furthermore, we consider concurrent transmissions to enhance channel use and hence increase network throughput. Consequently, the proposed protocol can outperform well-known MAC protocols in terms of E2ED, EC, PDR, TC, and Thpt [34]. The main contributions of this paper are as follows:

- Identify the most efficient MAC protocol approach for a pipeline topology over multi-hop for time-sensitive applications relative to the EE, COR, and FI.
- Develop an efficient MAC protocol that addresses the limitations of current MAC protocols, which is DAMAC, a novel scheduling algorithm that exploits the long propagation delay of acoustic channel to allow concurrent transmissions.
- Compare the simulation results of the proposed MAC protocol with other MAC protocols in terms of core metrics (e.g., EE, COR, FI).
- Identify the trade-offs of each MAC protocol approach in a pipeline topology over multi-hop.

The rest of this paper is organized as follows. In Section 2, we discuss the different techniques of MAC protocols. Section 3 presents articles related to the proposed MAC protocol and highlights the design characteristics examined in each paper. In Section 4, we describe the algorithm of our proposed MAC protocol. Section 5 provides the details of the performance evaluation and compares the proposed MAC protocol with several other MAC protocols. Section 6 concludes this paper and highlights some future directions.

## 2. Background

**Figure 1:**
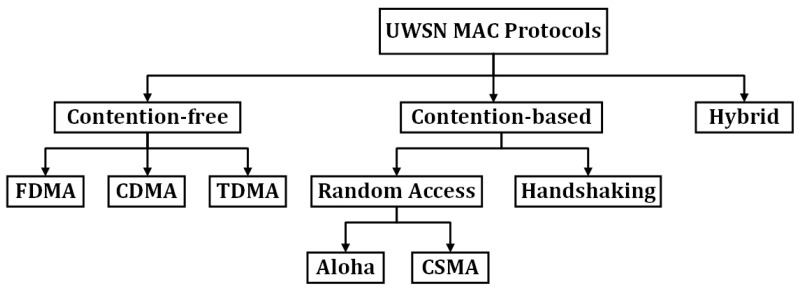
Classification of MAC Protocol Approaches [34] @ 2021 IEEE.

In this paper, we aim to enhance the performance of underwater communication by designing an efficient MAC protocol that can meet the Quality of Service (QoS) requirements of time-sensitive applications. To achieve this, we must analyze different MAC protocol approaches, which are classified into the categories of contention-free, contention-based, and hybrid [35,36,37,38]. Figure 1 illustrates the classification of the MAC protocol approaches.

**Figure 2:**
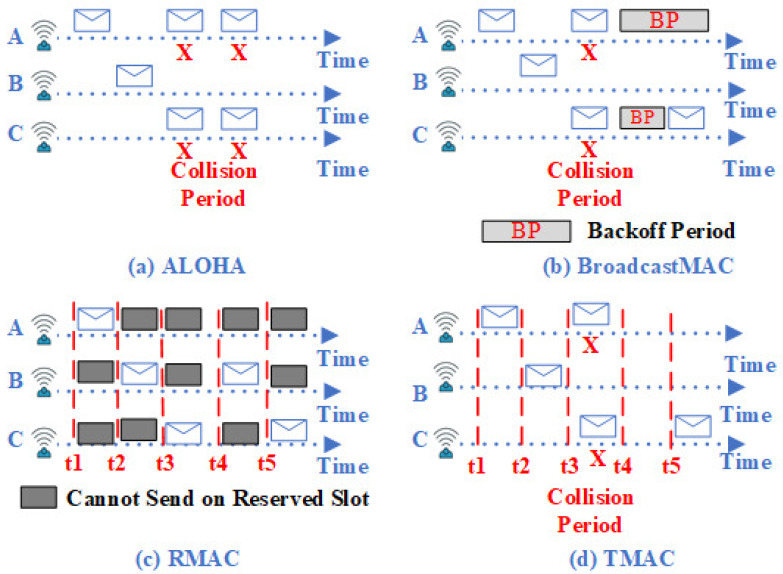
Operations of MAC Protocols [34] @ 2021 IEEE.

In contention-free (also known as schedule-based) MAC protocols, sensor nodes must reserve the channel to transmit packets between intended parties. The reservation can be achieved using a distinctive code, frequency, or time. This means that a sensor node can send packets using Code Division Multiple Access the (CDMA), Frequency Division Multiple Access (FDMA), or Time Division Multiple Access (TDMA) technique. For example, TDMA MAC (TMAC) protocol is considered a contention-free MAC protocol as it allows sensor nodes to transmit packets at a reserved time slot. In contention-based MAC protocols, sensor nodes can transmit packets using the random-access or handshaking technique. The random-access technique can be further classified into completely random or CSMA/CA. In the completely random approach, a sensor node can send packets at any time. A great example of a completely random-access approach is the Aloha protocol. In contrast, the CSMA/CA approach checks the channel status to start transmitting packets. If the channel is busy, backoff algorithm applied to avoid collisions between packets coming from neighboring nodes, otherwise packets can be transmitted toward the sink node. A great example of this type of approach is the BroadcastMAC protocol. The handshaking approach (also known as on-demand) avoids collision by requiring nodes to exchange multiple small control packets before starting to transmit data packets. The OPMAC protocol is a great example of the handshaking approach. The hybrid MAC protocols take advantage of both contention-free and contention-based approaches; one such hybrid protocol is Reservation-based MAC (RMAC). As can be observed from the above discussion, the contention-free approach can reduce collisions while the contention-based approach can minimize delay. This means that a contention-free approach is more appropriate to monitor non-time-sensitive applications while the contention-based approach is more appropriate for monitoring time-sensitive applications. In this study, we chose to evaluate different MAC protocol approaches to identify the most efficient for meeting the QoS requirements of oil/gas pipeline monitoring applications. Therefore, we selected the following MAC protocols, as each one represents a different MAC protocol approach. Figure 2 illustrates the operations of the MAC protocols that were used in this study.

### 2.1. Aloha

Aloha [39] is a contention-based random-access protocol that does not use CSMA/CA. Instead, it allows sensor nodes to transmit packets at any time a node has data to send. The simplicity of this protocol comes at the cost of increased numbers of collisions and decreased network performance.

### 2.2. BroadcastMAC

BroadcastMAC [39] is a contention-based random-access protocol that uses CSMA/CA, where a sensor node can transmit packets if the channel sensed is free, otherwise it backs-off. This protocol resolves the issue of Aloha by sensing the channel prior to transmitting packets, and therefore reducing the number of collisions and improving network performance. However, hidden-terminal, spatio-temporal uncertainty, and near-far problems can still exist.

### 2.3. Reservation-Based MAC (RMAC)

RMAC [40] is a hybrid-based MAC protocol that uses Time Division Multiple Access (TDMA) plus the handshaking approach to reserve a time slot. The aim of this protocol is to minimize energy consumption while avoiding collisions. To accomplish this, each sensor node randomly selects its own schedule, which helps to reduce the amount of energy waste during idle and overhearing periods [20,23]. However, to achieve a more collision-free network, this protocol allows only a single sensor node to transmit packets at any time slot throughout the network, which increases delay.

### 2.4. TDMA MAC for Underwater Networks (T${}_{u}$-MAC)

T${}_{u}$-MAC [39] is a contention-free MAC protocol that permits sensor nodes to send packets at the beginning of a reserved time slot. In this protocol, a sensor node must first transmit the Request-to-Send (RTS) packet to the intended receiver. Once the sensor node receives the Clear-to-Send (CTS) packet from the intended receiver, the sensor node then waits until the CTS packet propagates throughout the nodes within its transmission range and then transmits data packets. Due to the long propagation delay in UASNs, this protocol may experience collisions as it allows multiple sensor nodes that are out of range of one another to transmit packets, which can cause the hidden-terminal problem.

## 3. Related Work

In this paper, we classify underwater MAC protocols into contention-free (i.e., schedule-based), contention-based (i.e., reservation-based), and hybrid. Contention-free MAC protocols reserve a shared channel for a specific node based on frequency, code, or time [34]. Contention-based MAC protocols are further classified into random access and handshaking-based. Random-access protocols allow a node to transmit packets randomly. In contrast, handshaking MAC protocols require that nodes exchange control packets (e.g., RTS, CTS) prior to data transmission. Hybrid MAC protocols take advantage of contention-free to avoid collisions while relying on contention-based to support time-sensitive applications.

### 3.1. Contention-Free MAC Protocols

Sivagami and Manickam [41] proposed a Cluster-Based MAC (CBMAC), which is a contention-free protocol that employs TDMA to schedule the transmission time of each node by the Cluster Head (CH). This protocol aims to avoid collisions and reduce end-to-end delay using a duty-cycle mechanism to assign the available slots to cluster members based on their requests. Cluster members with no data to transmit can skip their slot at which point the CH assigns a new schedule to each member. The CH can build a conflict-free map, which allows nodes to transmit multiple packets concurrently. Although Distributed On-demand Schedule (DOS) [42] can transmit only a single transmission at a time, CBMAC allows multiple transmissions simultaneously within a cluster’s domain. The results show a significant improvement in terms of energy consumption, delay, and PDR compared to TDMA protocol. However, when CBMAC uses TDMA, some packets may be dropped. Hence, the source node must wait for its schedule to retransmit these lost packets, which can increase the delay. Furthermore, although CBMAC guarantees no collision within a cluster, collisions may exist between clusters. Thus, all types of single channel collisions may occur between clusters. This means that CBMAC may consume higher EC and hence shorten the network lifetime. In addition, using TDMA will increase end-to-end delay and fail to meet the requirements of time-sensitive applications. Moreover, due to the sparse node deployment and lack of time synchronization, underwater nodes that are farther away from the sink node may have lower opportunity to reserve the channel compared to closer nodes. This may waste network resources and further shorten network lifetime.

### 3.2. Contention-Based MAC Protocols

To deploy Slotted Floor Acquisition Multiple Access (S-FAMA) [43] in denser networks, Qian et al. [44] proposed RTS Competition S-FAMA (RC-SFAMA) to solve the low throughput and long propagation delay when two nodes send RTS packets simultaneously. In legacy S-FAMA, when multiple nodes concurrently send RTS packets, all nodes must defer their transmissions, which requires all nodes to wait. This mechanism degrades the throughput as it requires nodes to wait with no data transmission during the random backoff period. On the other hand, RC-SFAMA requires multiple RTS packets to compete to reserve the channel, and the winner transmits its data packets. RC-SFAMA is well-suited for dense networks because it improves the throughput and extends the network lifetime compared to S-FAMA. However, for a large-scale network, the throughput decreases as the load increases, which will also increase the delay. Using the random number generator to determine the winner may increase the delay, which is problematic in time-sensitive applications and cases where nodes with urgent data to transmit have a lower number than other competitor nodes. Similar to S-FAMA, RC-SFAMA may result in low network throughput and high energy consumption due to the spatio-temporal uncertainty and near-far problems. Relative to our proposed MAC protocol, the RC-SFAMA allows multiple nodes to compete and only a single node can reserve the channel, which results in a low EE and high COR due to the amount of control packets that must be exchanged to reserve a shared channel. Ultimately, RC-SFAMA consumes higher network resources and shortens the network lifetime; hence, it may not meet the QoS requirements of time-sensitive applications.

The traditional handshaking MAC protocols, such as S-FAMA [43], Multi-session FAMA (M-FAMA) [45,46], Multiple Access with Collision Avoidance Adaptive Packet Train (MACA-APT) [28], and MACA-based Power Control (MACA-PC) [35], employ RTS/CTS packets to reserve a channel. The RTS packet is used to request channel reservation, while the CTS packet replies to a received RTS packet. Dou and Peng [47] proposed an On-demand Pipelined MAC (OPMAC) protocol for time-sensitive applications. OPMAC is a contention-based MAC protocol that employs the RTS/CTS mechanism to reserve the channel and enable concurrent transmissions while the channel is idle. As underwater environment changes over time, OPMAC can support different network topologies, on-demand data traffic and on-demand path selections. This technique reduces delay by minimizing the number of control packets that must be exchanged between the intended parties. The proposed work changed the function of the CTS packet and added a new control packet called Data Acknowledgment (DACK). The CTS packet is used to reply to the previous hop and used as an RTS packet to the next relay hop. Similarly, DACK is used to acknowledge the previous node and transmit the data packets to the next node. The handshaking mechanism in the proposed work reduces the control packets’ overhead. Therefore, it reduces delay and energy consumption. OPMAC addresses the collisions to data packets within the transmission range, but control packets could still collide with ongoing data packets. In addition, hidden-terminal and exposed-terminal problems are not addressed by OPMAC, which may severely reduce network performance. This means that OPMAC consumes higher energy per byte and requires high COR due to exchange of control packets. Therefore, to enhance the performance of OPMAC, it is necessary to address all types of collisions and allow a packet train of data to be transmitted concurrently. Moreover, further enhancement can be achieved by reducing the amount of control packets by allowing multiple nodes that share control packets to reserve a channel and hence reduce the COR and energy consumption.

**Figure 3:**
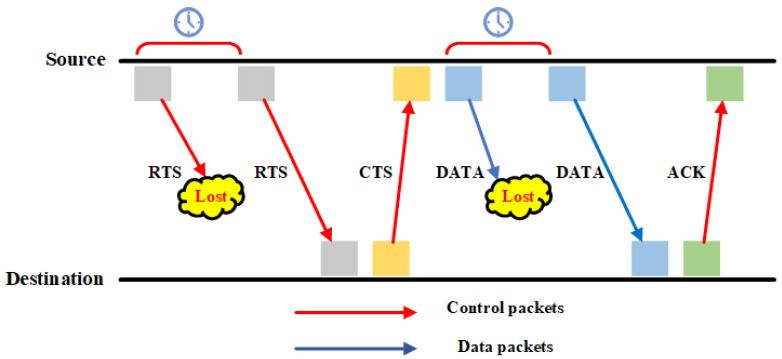
Retransmit Lost Control and Data Packets.

Due to the characteristics of acoustic channel, packets can be lost and must be retransmitted. When packets are lost in S-FAMA [43], M-FAMA [45,46], MACA-APT [28], MACA-PC [35], and OPMAC [47], the source must retransmit the lost packets via any data channel as illustrated in Figure 3. This process increases the end-to-end delay. To reduce the time taken to retransmit lost packets, Kim and Cho [48] proposed a cooperative Automatic Repeat Request MAC (ARQ-MAC) protocol. This protocol introduces one control packet to define the cooperators between source and destination. Furthermore, to transmit packets, a node first sends RTS to the destination. Then, nodes overhearing the RTS packet send a reply with a Request-To-Cooperate (RTC). The source selects the best cooperators based on the shortest path. Once the source receives the CTS packet from the destination, the source transmits data through the preferred cooperators. The great advantage of this protocol is that if packets are lost or an incorrect packet is received, the destination requests retransmission from the closest cooperator. Furthermore, to request retransmission from a cooperator, the destination sends a Negative ACKnowledgment (NACK) to the closest cooperator. If the destination fails to receive the missing packets from that closest cooperator, it then sends NACK to the next closest cooperator, and so on, until it receives the missing packets. This technique improves network throughput, reduces energy consumption, and ultimately reduces the end-to-end delay. However, end-to-end delay and network throughput would be further improved if ARQ-MAC avoided collisions to control and data packets and allowed concurrent transmissions simultaneously. Since ARQ-MAC allows only a single node to reserve a shared channel, this results in low EE and high COR by consuming more energy to transmit control packets. Moreover, ARQ-MAC must rely on the 3-D network architecture to minimize the amount of power that is required to transmit collected data between intended parties.

### 3.3. Hybrid MAC Protocols

An Improved Multi-Hop enabled Energy Efficient MAC (IMHEE-MAC) protocol has been proposed by Mozumder et al. [49] to solve the issues of MHEE-MAC protocol regarding control packet collisions in multi-hop networks. MHEE-MAC protocol uses two phases to avoid collisions, which requires visiting the time slot several times. IMHEE-MAC relies on a single reservation mechanism to avoid collisions and hence reduces energy consumption. Local nodes are assigned with a random priority number such that the higher number reserves the channel at the local network. A node with a high priority number from a different network transmits an RTS in the next time slot. The remaining nodes are kept in sleeping mode. The proposed work avoids local collision and hidden collision domains, which extends the network lifetime and provides high throughput compared to MHEE-MAC and S-FAMA [43] protocols. The network lifetime is also extended as the IMHEE-MAC protocol avoids collisions, and some nodes are active while others are in sleep mode. Collisions may occur if both nodes from different domains have the same priority number and reach the destination at the same time (hidden-terminal problem). Another issue is that as IMHEE-MAC assigns random priority numbers, it is possible that higher critical data packets may have a lower random priority number than less critical data packets. Hence, using IMHEE-MAC to transmit high critical data may create high E2ED and EC. Another limitation of IMHEE-MAC is that it relies on the traditional handshake where each node must send control packets to reserve a channel. To resolve this issue, IMHEE-MAC can reduce the control packets by allowing fewer numbers of control packets to represent a group of underwater nodes. In addition, IMHEE-MAC should allow multiple nodes to transmit packets concurrently by using the long propagation delay of UASNs to improve the EE, COR, FI, and other key performance metrics.

The contention-free protocols can meet the requirements of non-time-sensitive applications with low throughput and low energy consumption. In contrast, the contention-based protocols trade energy consumption for low delay high throughput, and high channel use. Therefore, Gorma and Mitchell [50] proposed a hybrid approach called Combined Free/Demand Assignment Multiple Access (CFDAMA) to take advantage of both protocol types. In CFDAMA, there is a sink node at the surface level and several nodes laid on the seabed. By default, a seabed node requests to send data using the contention-based approach. The surface node replies to the requested packets on the First-In First-Out (FIFO) basis. Based on individual requests, the surface node schedules each node with a number of slots using TDMA. The surface node switches between contention-based and contention-free contingent on the status of the reservation request table. Once all requests have been queued, the surface node switches to contention-free approach by assigning slots in a round-robin fashion. It takes the top address on the free assignment table followed by the next one, and so on. CFDAMA minimizes delay while maximizing channel use compared to contention-free and contention-based protocols. Although CFDAMA yields better results when compared to the TDMA protocol, it may suffer from collisions due to Triple Hidden-Terminal (THT) problems. Furthermore, as CFDAMA does not consider collisions to control packets, this may result in high E2ED, EE, COR, and low FI to those nodes that use the contention-based approach to reserve a data channel. Similar to ARQ-MAC [48], CFDAMA should rely on 3-D network architecture and allow concurrent transmission to enhance underwater communication in terms of the EE, COR, FI, and other key performance metrics.

In response to ERCA-MAC [51] and IMHEE-MAC [49], Alfouzan et al. [30] proposed an Efficient-Depth MAC (ED-MAC) protocol, which is a contention-based protocol that aims to meet the requirements of energy-critical applications. Each node in the network is assigned a time slot(s) to reserve the channel. The channel is then broken down into time slots, and each slot is further divided into sub-slots. The proposed work also addresses the spatio-temporal uncertainty and hidden-terminal problems to reduce collisions and retransmissions. A random mechanism is used to select the sub-slots to avoid collisions. Additionally, starting from higher to lower depth, each node broadcasts its beacon packet to its one-hop neighbor. This mechanism helps one-hop neighbors to determine which slots have been reserved to avoid collisions as well as to schedule wakeup and sleep times. Nodes can be in sleep mode when there is no data to send to conserve energy. The results show significant improvements in terms of energy consumption, PDR, and fairness across the network with various traffic rates and numbers of nodes. However, as ED-MAC does not address collisions to beacon packets, network performance can be degraded. Hence, network resources may not be used in the most efficient manner and ultimately may not be able to meet the QoS requirements of target application. Furthermore, similar to the issue with IMHEE-MAC [49], ED-MAC operates based on the traditional handshake procedure. This results in high COR and hence consumes higher EE. To further enhance the performance of ED-MAC, its procedure should be modified in such a way that a single control packet can serve multiple underwater nodes in reserving a channel to enhance the EE and COR. In addition, Ed-MAC should allow multiple nodes to transmit packets to a shared destination node simultaneously.

As observed from the discussion presented in the literature review, different target underwater applications require different designs of MAC protocols to meet their requirements. Table 2 summarizes how each MAC protocol type can be designed to enhance the performance of underwater communication. Different MAC protocols outperform on selected metrics at the cost of other key performance metrics. Most of these MAC protocols focus on improving energy consumption in a specific network topology by reducing the total number of collisions, but at the cost of lower throughput, higher E2ED, higher COR, and higher EE. Hence, a MAC protocol may outperform several existing MAC protocols on a specific network topology even though it will not give the same result in a different network topology. Therefore, since different applications have different QoS requirements, designing a MAC protocol suitable for UWSN applications must give due consideration to these requirements [20,31,32]. However, the literature review reveals that there is no single MAC protocol that dominates in all metrics for all underwater applications [20,31]. Since ED-MAC and APD-TDMA rely on the TDMA and handshake-based approaches, transmitting packets to intended parties results in high E2ED, EE, COR, and low FI—because farther nodes may not have equal opportunity to reserve the shared channel compared to a node that is closer to the sink node. Similarly, the DAP-MAC uses the random-access approach to transmit packets, but concurrent transmission only occurs at the beginning of time slot. This results in high E2ED and EC, because underwater nodes must wait for the next round to transmit collected data. Therefore, in this research we aim to design a MAC protocol that employs the random access-based approach for monitoring applications of the oil/gas pipeline industry in such a way that multiple nodes can transmit collected data at any time. This can be achieved by using the long propagation delay between underwater nodes to schedule data properly and hence improve the performance metrics relative to the EE, COR, and FI.

In this section, we discussed different MAC protocol approaches where each approach is suitable for a specific environment, network topology, and application. The existing MAC protocols address collision issues at the cost of higher delay. In particular, most of the previously proposed MAC protocols rely on the handshake-based approach where a node must go through a four-way handshake (e.g., RTS, CTS, DATA, ACK) to complete one cycle of transmitting data packets [7]. Taking another approach, many MAC protocols enhance the performance of underwater communication by using TDMA, slotted-TDMA, or slotted-Aloha to transmit packets between intended parties. Using TDMA or dividing the time into several slots, allows nodes only to transmit packets at the beginning of time slots, which causes excessive delay for time-sensitive applications. Due to the characteristics of underwater acoustic channel, using these approaches is not suitable for the requirements of time-sensitive applications as they result in high E2ED [60]. Table 3 highlights the characteristics and limitations of different MAC protocols.

To identify the key performance metrics that should be used for evaluation, we reviewed all the MAC protocols presented in this paper. Table 4 summarizes which metrics are used in other research papers. This table shows which metrics researchers must evaluate to achieve a complete analysis of their proposed MAC protocol. Due to the unique characteristics of the underwater environment and other issues, underwater applications focus on different performance metrics, but improving the key performance metrics often comes at a cost to other metrics. Therefore, researchers must be aware of the trade-offs of their proposed MAC protocols. In Table 4, we use a check mark to represent that a research paper considered the specific performance metric for evaluation. This table shows that there are eight common performance metrics (i.e., E2ED, EC, PDR, EE, TC, Thpt, COR, and FI). In our earlier work [34], we evaluated the E2ED, EC, PDR, TC, and Thpt. To provide a complete analysis, it is necessary to conduct further analysis regarding EE, COR, and FI.

## 4. Methods

**Figure 4:**
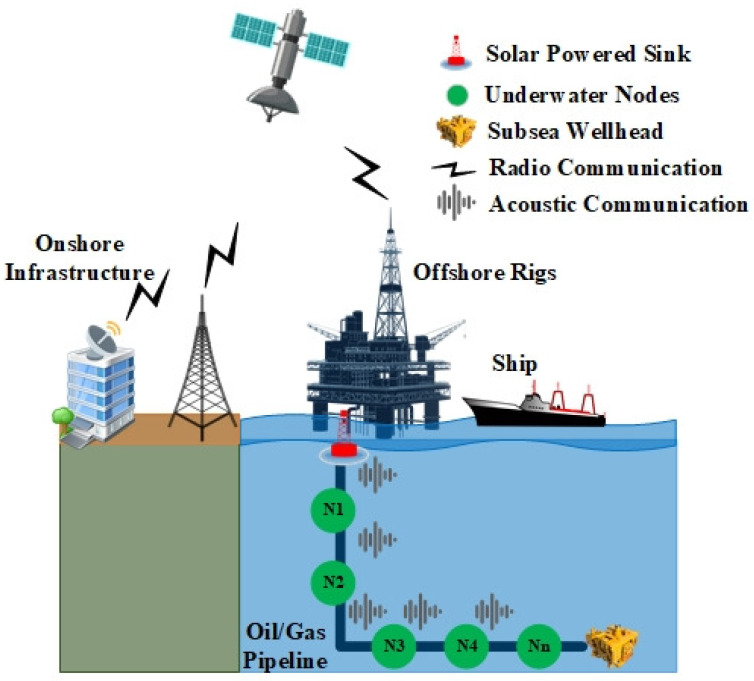
Simulation Network Topology [34] @ 2021 IEEE.

In this research, we only consider applications that consist of non-mobile underwater sensors under varying network loads that are designed to monitor oil/gas pipeline in shallow water. Oil/gas exploration can exist in shallow water, deep water, and ultra-deep water. The major difference between these three conditions are, obviously, the depth of the water. Specifically, shallow water, deep water, and ultra-deep water denote up to 125 m, 125–1500 m, and above 1500 m, respectively [1,61,62]. However, the majority of global crude oil production occurs in shallow water [61,62]. Offshore oil/gas rigs must constantly monitor the status of the pipeline to avoid disaster. These pipelines do not move and can last for years, thus non-mobile nodes can be used to help operators monitor the status of the underwater environment. In addition, due to the large target area, underwater nodes must be organized in a 3-D network architecture to minimize the power consumed in transmitting packets toward the sink node and hence reduce the EC. Underwater modems have different capabilities; for our research, we chose an underwater modem that is designed for shallow water applications and that consumes lower energy to extend network lifetime. Specifically, in this study we rely on the LinkQuest UWM1000 underwater modem. As shown in Figure 4, the network consists of several underwater sensors organized in an ad hoc form, where the sensed information is relayed from underwater nodes to reach the sink node at the surface level. Some assumptions are made in this experiment, which are as follows. First, underwater sensor nodes are operated in half-duplex mode, which means underwater nodes cannot send and receive packets concurrently. Second, the sensor nodes are located more than one-hop from the sink node except node one. Third, the simulation results show the network performance from underwater sensors to the sink node at the surface level.

The proposed DAMAC protocol is a delay-aware MAC protocol. To enhance channel use, the proposed MAC protocol eliminates the handshaking packets and uses concurrent transmission on the same time slot. To reduce the collision rate, the proposed MAC protocol schedules data transmission based on the long propagation delay. Therefore, a node transmits its data packets based on a pre-defined schedule. If the sink node receives the transmitted data, it will send an acknowledgment to inform the sensor nodes that it has received the data packets successfully. If the sensor nodes receive acknowledgment that the transmitted data packets have been successfully received, they can then send the next data packets on the next time slot. Otherwise, if no acknowledgment is received, the sensor nodes will retransmit the lost data packets.

To set up the waiting time, we divide the underwater nodes into two groups. Underwater nodes that belong to Group One will transmit their data packets while underwater nodes that belong to Group Two are in sleep mode to avoid wasting energy. In this way, the long propagation delay between nodes in the different groups helps to allow concurrent transmission and enhance network performance. At the next data transmission schedule, underwater nodes that belong to Group Two transmit their data packets while underwater nodes that belong to Group One are in sleep mode. This process repeats until either the underwater nodes in both groups do not have any more packets to send or the simulation time ends. The algorithm of the proposed MAC protocol is shown in Algorithm 1. Table 5 defines the meaning of each symbol in Algorithm 1.

Based on Algorithm 1, we can compute the time complexity to find the upper bound and lower bound of the proposed algorithm. DAMAC protocol relies on the total number of source nodes. As we increase the total number of source nodes, the total number of received packets at the sink node increases as well. However, the function runs in constant time to its input. Therefore, the upper bound of the DAMAC protocol is equal to f(n) = 91 ⇒f(n) = O(1). Similarly, the lower bound of DAMAC protocol is equal to f(n) = Ω(1).
**Algorithm 1:** DAMAC Protocol Process
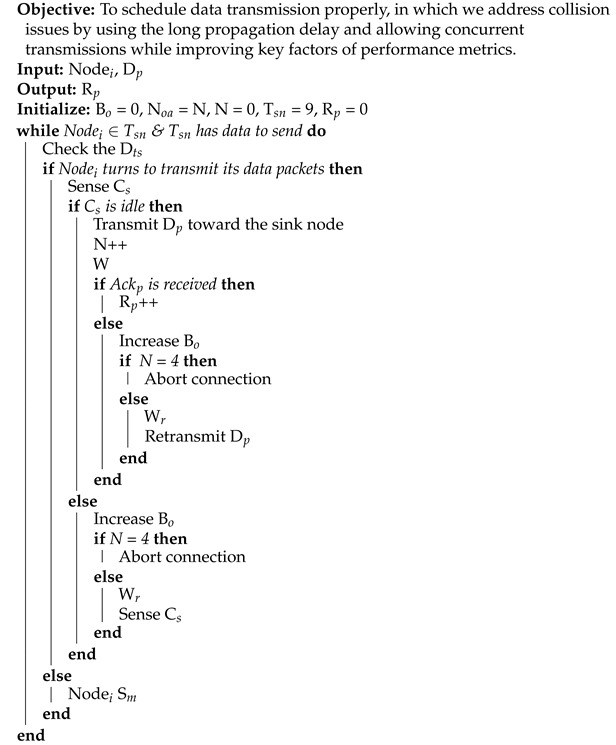


In this paper, we aim to develop a MAC protocol that uses an efficient scheduling algorithm where each node senses the shared channel and considers the long propagation delay between sensor nodes for data transmission. Based on the long propagation delay, we can resolve the spatio-temporal problem. In this case, we can allow multiple nodes that share a common channel to transmit packets concurrently to increase network use. In addition, the proposed MAC protocol can avoid collisions if the propagation delay between sensor nodes is long enough that ongoing packets do not collide with other packets coming from other nodes.

## 5. Performance Evaluation

In this study, we focus on monitoring oil/gas underwater pipeline in shallow water. The underwater network is deployed with 3-D network architecture, where numbers of underwater nodes are laid at different depths and a single sink node is placed at the water’s surface. The underwater network is broken down into onshore infrastructure and offshore infrastructure. The offshore infrastructure consists of sensor nodes, a sink node, offshore rigs, and a ship. In contrast, the onshore infrastructure consists of the data center, where collected data can be monitored, as well as a refinery. The operation of the oil/gas industry is divided into three sectors, which are: upstream, midstream, and downstream [63,64]. The main objective of the upstream sector is to search for areas where oil/gas materials may exist and then extract them. Once the raw materials have been extracted, they must be stored and then moved to a refinery. The midstream sector is responsible for the storage and transport of the raw materials. After the raw materials have been processed in a refinery, they are considered converted into a product. Turning the raw materials into a product is the responsibility of the downstream sector. The oil/gas industry uses offshore rigs to look for raw materials and extract them. In this study, we focus on monitoring this upstream sector by deploying underwater sensors at different depths and a single sink node at the surface level. Oil tankers have barrels to store raw materials and transport them to a refinery. The refinery is the onshore infrastructure that represents the downstream sector.

Underwater applications are classified into time-sensitive and non-time-sensitive applications. Time-sensitive applications focus on minimizing delay while non-time-sensitive applications aim to reduce energy consumption. The oil/gas industry requires that any potential disaster be detected with minimal delay and maximum PDR, which makes it a time-sensitive application. In this study, we investigate which MAC protocol technique can meet the QoS requirements of this type of application. To achieve this, we must study different MAC protocol techniques. Therefore, we evaluate the following five different MAC protocols: TDMA MAC for underwater networks (T${}_{u}$-MAC) [39], Aloha [39], BroadcastMAC [39], Reservation-based MAC (RMAC) [40], and OPMAC [47]. Each one of these MAC protocols relies on a different MAC protocol technique. In particular, T${}_{u}$-MAC relies on TDMA, which is a contention-free MAC protocol. Aloha is a completely random protocol, which makes it a great example of a contention-based random-access MAC protocol. Similarly, BroadcastMAC is another example of contention-based random-access MAC protocol, but one that uses CSMA to transmit packets between intended parties. RMAC relies on TDMA and handshaking techniques, which makes it a great example of a hybrid MAC protocol. OPMAC depends on only the handshaking technique and is considered to be an accurate example of a contention-based handshake MAC protocol.

### 5.1. Simulation Setup

We used Aloha, BroadcastMAC, RMAC, T${}_{u}$-MAC, and OPMAC protocols using the Aqua-Sim simulator, which is built on top of the Network Simulator 2 (NS-2) [39,65]. Aqua-Sim is a well-known tool for the simulation of UWSNs. As shown in Figure 4, the underwater network operates as an ad hoc network where each node transmits collected data to its neighboring nodes to reach the sink node. This means that underwater nodes send collected data to the sink node in multi-hop fashion. In this study, we assume that all nodes are static and deployed randomly. The amount of energy consumption depends on the energy model where each underwater modem may have different energy consumption values for sending, receiving, and idle states. The lower the energy consumption values, the longer the network lifetime. Therefore, we relied on the energy model of the LinkQuest UWM1000 acoustic modem. Table 6 provides the details of all simulation parameters used in this study.

### 5.2. Performance Metrics

We analyzed the simulation results relative to energy efficiency, communication overhead, and fairness of the network. A brief description of these performance metrics are given as follows:

1. Energy Efficiency (*EE*) is the sum of energy consumed by all nodes divided by the total number of received bytes at the sink node and multiplied by the Packet Size (PS) [30,44]. *EE* is measured in Joules per Byte (*J/B*). A lower energy consumption per received byte is always preferable to achieve higher *EE*. This means that as when we spend lower energy to receive bytes, the *EE* is higher and hence the network lifetime is longer. *EE* can be calculated using:
  (1) $$\begin{array}{c} EE\;\left[J/B\right]=\;\frac{EC\;}{TR_{s_{n}}\times PS\;} \end{array}$$
2. Communication Overhead Ratio (*COR*) can be defined as the total number of link layer packets divided by the total number of all packets [55].
  (2) $$\begin{array}{c} COR\;=\;\frac{T_{mac}}{T_{all}}\times 100 \end{array}$$
  where $T_{mac}$ = total number of MAC layer packets; $T_{all}$ = total number of all packets generated by all nodes in the network.
3. Fairness Index (*FI*) is a critical performance metric of MAC protocol to ensure fairness among nodes that use a shared channel [30,58,66]. Fairness ensures that all nodes transmit the same amount of data packets to the sink node. The index value of *FI* affects the network survival time of UWSNs. To evaluate fairness of the network, we use Jain’s *FI* [18,30], which can be defined as follows:
  (3) $$\begin{array}{c} FI\;=\;\frac{{\left(\sum x_{i}\right)}^{2}\;}{\left(n\times \sum x_{i}^{2}\right)} \end{array}$$
  where $x_{i}$ = throughput of node *i*; *n* = total number of nodes in the network. The range of index value lies between 0 and 1. Zero indicates the protocol has a poor performance. One indicates the protocol has an optimal performance. In other words, “0” indicates that the total number of received packets is not the same from all underwater nodes. In contrast, as the index value approaches “1,” this indicates that the total number of received packets is almost the same from all underwater nodes.

To enhance network performance, nodes wake up and sleep in a repeating cycle. They are awake in particular slots to transmit or receive packets and asleep during the other time slots according to the data schedule. Every slot is reserved for a certain number of nodes to transmit and receive packets. The length of each slot is fixed and equal to E2ED plus a short guard time to avoid collisions at the receiver side. Each node knows its own schedule to transmit its data packets as well as its one-hop neighbor’s schedule. This known schedule determines when each node should wake up to transmit its own data packets or to receive packets from neighboring nodes. This schedule helps to avoid collisions between data packets coming from different nodes, aligns the sleep schedule to the data schedule, and hence improves the network’s performance, in terms of E2ED, EC, PDR, TC, and Thpt [34]. To provide a full analysis of the proposed protocol, we focused on evaluating the impact of all MAC protocols in terms of EE, COR, and FI.

1. *EE*: This is an important factor for determining how efficient a MAC protocol is in terms of energy consumption compared to the number of received data packets. Optimally, the less energy needed to receive packets, the longer the network lifetime. In other words, lower value of *EE* means that a MAC protocol is more efficient as it consumes less energy to receive packets and vice versa.
2. *COR*: From Equation (2), $T_{mac}$, this is the sum of packets generated by source nodes. The value of $T_{mac}$ can determine the performance of a MAC protocol by computing the total number of packets that must be generated by source nodes to transmit control and data packets. To evaluate a MAC protocol in terms of efficiency, the overhead should be kept to a minimum. Increased overhead means more packets must be generated, which results in higher E2ED and EC. Therefore, lower overhead is preferable to meet the QoS requirements of an application. $T_{mac}$ can be calculated using:
  (4) $$\begin{array}{c} T_{mac}=\left(\lambda \times S_{t}\right)-\left(\left(\lambda \times S_{t}\right)\times n\right) \end{array}$$
  From Equation (2), we also compute $T_{all}$. $T_{all}$ refers to the total number of all packets generated by all nodes in data link and network layers. Therefore, $T_{all}$ is the sum of $T_{mac}$ and the total number of routing packets in the network $\left(T_{r_{o}}\right)$. $T_{all}$ can be calculated using:
  (5) $$\begin{array}{c} T_{all}=T_{mac}+T_{ro} \end{array}$$
  From Equation (4), $T_{ro}$ is the sum of the total number of routing packets in the network multiplied by the total number of links from the source nodes to the sink node. Network topology plays a critical role in meeting an application’s QoS requirements. As we increase the hop length between source nodes and the sink node, the E2ED and EC increases as well and vice versa. $T_{ro}$ can be calculated using:
  (6) $$\begin{array}{c} T_{ro}=\sum_{i=1}^{n}TS\times T_{l}\times 2 \end{array}$$
  where $T_{l}$ = total number of links from the source nodes to the sink node. The number of links depends on the location of the source nodes.
  Substituting Equations (4) and (6) into Equation (2) to compute *COR*:
  (7) $$\begin{array}{c} COR=(\frac{\left(\lambda \times S_{t}\right)-\left(\left(\lambda \times S_{t}\right)\times n\right)}{\left(\left(\lambda \times S_{t}\right)-\left(\left(\lambda \times S_{t}\right)\times n\right)+\left(\sum_{i=1}^{n}TS\times T_{l}\times 2\right)\right)}) \end{array}$$
3. *FI*: From Equation (3), substitute $x_{i}$ from Equation (3):
  (8) $$\begin{array}{c} \frac{\left(\left(\sum_{i=1}^{n}\;TR_{i}\right)\times 8\right)}{\left(S_{t}\times 1000\right)}FI=\frac{{\left(\sum_{i=1}^{n}\left(\frac{\left(TR_{i}\times 8\right)}{\left(S_{t}\times 1000\right)}\right)\right)}^{2}}{n\times {\left(\sum_{i=1}^{n}\frac{\left(TR_{i}\times 8\right)}{\left(S_{t}\times 1000\right)}\right)}^{2}} \end{array}$$

### 5.3. Results and Analysis

**Figure 5:**
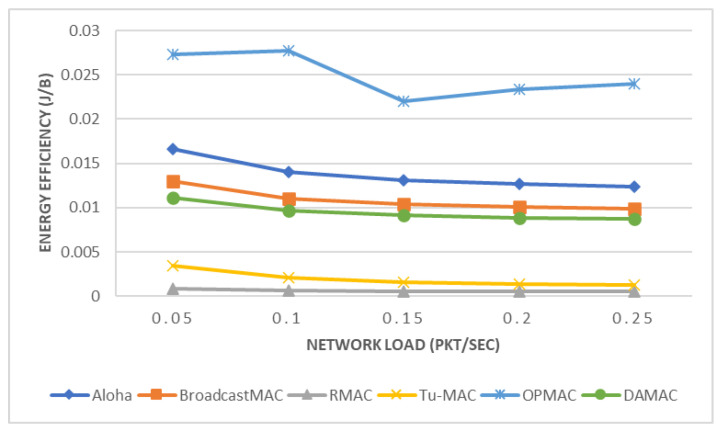
Energy Efficiency.

**Figure 6:**
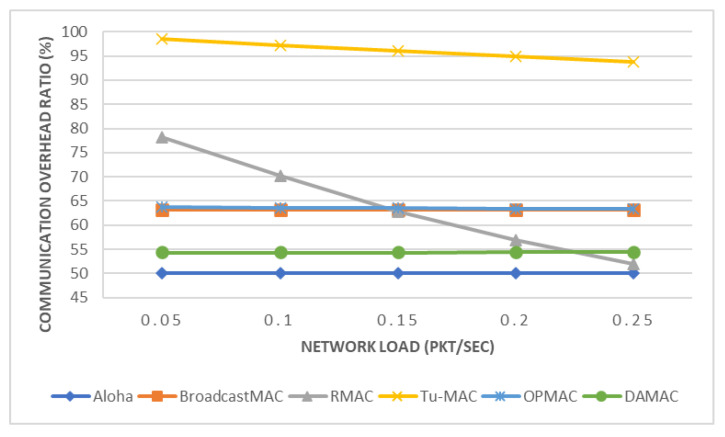
Communication Overhead Ratio.

**Figure 7:**
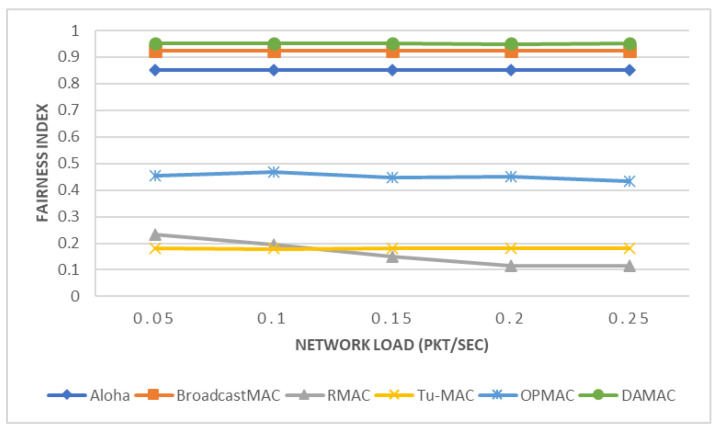
Fairness Index.

In this section, we present the performance of the DAMAC protocol under varying network loads and compare our proposed protocol with five different MAC protocols: T${}_{u}$-MAC, Aloha, BroadcastMAC, RMAC, and OPMAC. Network load plays an important role in enhancing the performance of UASNs [67]. In particular, we compare these five protocols in terms of EE (see Figure 5), COR (see Figure 6), and FI (see Figure 7). For the purposes of this experiment, underwater nodes relied on a multi-hop network where data packets are transmitted toward the sink node through relay nodes.

In Figure 5, the EE of Aloha [39], BroadcastMAC [39], RMAC [40], T${}_{u}$-MAC [39], OPMAC [47], and DAMAC protocols is inversely proportionate to the network load. This means that as we increase the network load, lower energy consumption per byte and hence researchers are recommended to use higher network load to improve EE. Aloha, BroadcastMAC, and DAMAC protocols offer much higher EE compared to RMAC, T${}_{u}$-MAC, and OPMAC protocols, due to their higher PDR. Moreover, the DAMAC protocol offers better EE compared to the Aloha, BroadcastMAC, and OPMAC protocols due to its being schedule-based. In particular, the DAMAC protocol achieves higher EE by 29–33%, 12–14%, and 59–65% compared to Aloha, BroadcastMAC, and OPMAC protocols, respectively. This means that DAMAC consumes lower energy per byte and hence can better extend network lifetime in comparison with the Aloha, BroadcastMAC, and OPMAC protocols. In contrast, the DAMAC protocol offers lower EE by 1160–1612% and 220–608% compared to the RMAC and T${}_{u}$-MAC protocols, respectively. The main reason for this is that DAMAC protocol receives a much higher number of bytes compared to both the RMAC and T${}_{u}$-MAC protocols. This means that DAMAC consumes a higher amount of energy per byte relative to the RMAC and T${}_{u}$-MAC protocols at the cost of increased PDR. Based on the above results, the DAMAC protocol provides higher EE as compared to Aloha, BroadcastMAC, and OPMAC while resulting in lower EE when compared to the RMAC and T${}_{u}$-MAC protocols. This means that DAMAC protocol outperforms contention-based MAC protocols, but that contention-free and hybrid MAC protocols can offer the highest EE but one that comes at the cost of low PDR.

We show in Figure 6 that the communication overhead of the Aloha [39], BroadcastMAC [39], OPMAC [47], and DAMAC protocols remains the same regardless of network load. In contrast, the communication overhead of RMAC [40] and T${}_{u}$-MAC [39] protocols is indirectly proportionate to the network load. This indicates that the network load either does not impact the COR or reduces COR as network load increases and thus researchers are strongly encouraged, when transmitting large number of packets, to use a high network load. The proposed MAC protocol requires fewer MAC packets to communicate between intended parties compared to the BroadcastMAC, RMAC, T${}_{u}$-MAC, and OPMAC protocols by about 14%, 4–30% (except at the highest network load), 42–45%, and 14–15%, respectively. The proposed MAC protocol achieves lower communication overhead compared to BroadcastMAC due to concurrent transmissions. DAMAC allows multiple packets to be transmitted from several nodes and hence fewer MAC packets are transmitted. RMAC and T${}_{u}$-MAC protocols result in high communication overhead as they both use control packets (e.g., RTS and CTS) to reduce collisions prior to data packet transmission. This indicates that the proposed MAC protocol can reduce the COR as compared to the BroadcastMAC, RMAC, T${}_{u}$-MAC, and OPMAC protocols and hence requires lower energy consumption. Since underwater nodes are operated with limited battery, lower energy consumption is always preferable to extend the network lifetime. As the network load increases, the RMAC protocol will require less communication overhead, but at the cost of higher E2ED. Moreover, OPMAC requires less packet overhead compared to T${}_{u}$-MAC protocol as it reduces the number of control packets to reserve a channel. This means that using the OPMAC protocol can extend the network lifetime as compared to the T${}_{u}$-MAC protocol. The communication overhead of Aloha requires fewer packets compared to the proposed MAC protocol by about 9%, as this protocol transmits packets whenever the channel is available. Although Aloha achieves the lowest communication overhead, it results in high end-to-end delay, energy consumption, and total number of collisions, while achieving lower PDR and throughput compared to the proposed MAC protocol. To meet the QoS requirements of oil/gas pipeline monitoring applications, the results of this paper show that the proposed DAMAC protocol is the most appropriate protocol to support such time-sensitive applications.

In Figure 7, we observe that the fairness of the network of the Aloha [39], BroadcastMAC [39], T${}_{u}$-MAC [39], and DAMAC protocols is constant to the network load. This means that underwater nodes will have the same opportunity to access the channel and transmit collected data regardless of the network load. In these types of protocols, it is highly recommended to use a high network load to achieve lower COR and EE. In contrast, the fairness of the network of RMAC [40] protocol is inversely proportionate to the network load. This means that when using the RMAC protocol, some underwater nodes will have less opportunity than other nodes as the network load increases. Consequently, it is likely that an underwater node would not be able to transmit critical data during a disaster due to the operation of the RMAC protocol, which could hence increase the severity of total loss. Therefore, it is critical that a MAC protocol share network resources more efficiently to alert operators with minimal delay in case of incidents of disaster. The fairness of the network of OPMAC [47] protocol varies based on the given network load. Aloha, BroadcastMAC, and DAMAC protocols offer much higher fairness of the network compared to the RMAC, T${}_{u}$-MAC, and OPMAC protocols. This indicates that the Aloha, BroadcastMAC, and DAMAC protocols share network resources more fairly among the nodes compared to the RMAC and T${}_{u}$-MAC protocols. This is because of the competition to access the channel by exchanging control packets, which lowers the fairness. DAMAC protocol achieves the highest fairness of the network compared to other MAC protocols due to the schedule-based algorithm. This means that the DAMAC protocol can support oil/gas pipeline monitoring applications more efficiently in comparison to other well-known MAC protocols. In particular, DAMAC protocol outperforms Aloha, BroadcastMAC, RMAC, T${}_{u}$-MAC, and OPMAC protocols by about 12%, 3%, 307–726%, 429–430%, and 103–120%, respectively. Based on the above results, the Aloha, BroadcastMAC, and DAMAC protocols are more appropriate to support time-sensitive applications than the RMAC, T${}_{u}$-MAC, and OPMAC protocols.

## 6. Conclusions

In this paper, we developed a DAMAC protocol for a delay-sensitive application in UASNs. The proposed MAC protocol relies on a scheduling-based mechanism that allows concurrent transmission to enhance poor performance in the UASN setting. This study reveals that the random-access-based approach is considered the most appropriate MAC protocol to support a pipeline topology over multi-hop in terms of EE, COR, and FI. The DAMAC protocol addresses the limitations of existing MAC protocols by using an efficient scheduling algorithm that enhances underwater communication. Moreover, we analyzed each MAC protocol approach to highlight the trade-offs that exist with each. In particular, the Aloha, BroadcastMAC, and DAMAC protocols are considered more suitable to meet the QoS requirements of time-sensitive oil/gas pipeline monitoring applications. Although the T${}_{u}$-MAC and RMAC protocols offer higher energy efficiency, as compared to the Aloha, BroadcastMAC, OPMAC, and DAMAC protocols, they both receive a much lower number of bytes. This means that the T${}_{u}$-MAC and RMAC protocols cannot achieve maximum PDR and hence are not suitable for monitoring time-sensitive applications. In short, the proposed MAC protocol exhibits significant enhancements compared to other MAC protocols in terms of EE, COR, and fairness of the network, which makes it the most suitable one for time-sensitive monitoring applications. This study can also serve as a guide on how to meet the QoS requirements of target applications. Since this research focused on a static underwater large-scale network, further investigation is needed to evaluate the developed DAMAC protocol in a mobile network and/or a small-sale network. Furthermore, to expand the scope of this research, the developed DAMAC protocol should be evaluated in the targeting of different underwater applications.

## Figures and Tables

**Table 1:** Summary of UASN Issues and Requirements.

Issues	Summary
Collision rate [25,26,27]	The collision rate depends on the type of MAC protocol approach. Using the long propagation delay to schedule data packets properly can help in reducing the collision rate.
Communication overhead [28,29,30]	The communication overhead is related to how many packets are required to transmit data between intended parties. The MAC protocols that rely on the handshake-based approach result in high COR.
Energy consumption [21,22,23]	Scheduling data properly and allowing concurrent transmission can minimize energy consumption and hence extend the network lifetime.
QoS requirements [14,20,31,32]	Each MAC protocol approach has its benefits and limitations. Using the appropriate approach can help to meet the key performance metrics by factoring the unique needs of the target application.

**Table 2:** Summary of the Design Characteristics of UWSN MAC Protocols.

Propose	Year	Classification	Required Information to Reduce Collisions	Network Architecture	Network Topology	Mobility	Communication Transmission
CBMAC [41]	2016	TDMA	1-HN	3-D	C	No	MT
GC-MAC [26]	2019	TDMA	2-HN	3-D	C	No	MT
UW-SEEDEX [24]	2021	TDMA	2-HN	3-D	AH	Yes	ST
**Contention-based MAC Protocols**							
Modified-Slotted-Aloha [37]	2020	RA	1-HN	3-D	AH	No	ST
DAP-MAC [52]	2016	RA	1-HN	3-D	AH	Yes	MT
TARS [29]	2017	RA	1-HN	3-D	AH	Yes	ST
RC-SFAMA [44]	2015	H	NWTR	2-D and 3-D	AH	No	MT
MACA-PC [35]	2016	H	NWTR and NWIZ	2-D and 3-D	AH	No	MT
OPMAC [47]	2015	H	1-HN	3-D	AH	No	MT
ARQ-MAC [48]	2016	H	1-HN	2-D	AH	No	ST
ACP-CA [53]	2021	H	1-HN	3-D	AH	No	ST
PC-MAC [54]	2017	H	NWTR	3-D	C	Yes	ST
CPOR [55]	2017	H	NWTR	3-D	AH	No	ST
**Hybrid MAC Protocols**							
IMHEE-MAC [49]	2017	HB	NWTR	3-D	AH	No	ST
CFDAMA [50,56]	2017	HB	NWTR	2-D	AH	No	ST
ED-MAC [30]	2018	HB	1-HN	3-D	AH	No	ST
PB-MAC [57]	2017	HB	NWTR	4-D	AH	Yes	ST
HTCC [58]	2016	HB	NWTR	2-D and 3-D	AH	No	MT
APD-TDMA [59]	2018	HB	1-HN	3-D	C	Yes	ST

RA: Random Access, H: Handshaking, HB: Hybrid, 1-HN: 1-Hop Neighbors, 2-HN: Two-Hop Neighbors, NWTR: Nodes Within Transmission Range, NWIZ: Nodes Within Interference Zone, AH: Ad hoc, C: Cluster, ST: Single Transmission, MT: Multiple Transmissions.

**Table 3:** Summary of Characteristics and Limitations of MAC Protocols.

Protocols	Characteristics	Limitations
CBMAC [41]	Use a duty-cycle mechanism to schedule data properly.	Fixed duty-cycle mechanism may result in a long delay.
GC-MAC [26]	Avoids collision by assigning a unique time and color to each cluster.	Does not allow concurrent transmission from different clusters.
UW-SEEDEX [24]	Avoids collisions by exchanging the seed value of a node’s neighbor.	Requires each node to keep track of the schedule of other nodes.
Modified-Slotted-Aloha [37]	Introduces a new backoff technique and a buffer mechanism to minimize energy consumption.	Collisions may occur, and packets can only be sent at the beginning of the time slot.
DAP-MAC [52]	Exploits the long propagation delay to avoid collision.	Allows multiple transmissions only at the beginning of a time slot.
TARS [29]	Considers both environmental and non-environmental factors to improve network performance.	Hidden-terminal, Exposed-terminal, and Near-far problems could degrade network performance.
RC-SFAMA [44]	Introduces control packet competition to avoid collisions in control packets.	Uses random number generator that may result in high delay to urgent data.
MACA-PC [35]	Uses power level to alert neighbors about potential for collision.	Nodes defer transmission even if it would not cause collisions to their neighbors.
OPMAC [47]	Reduces the amount of control packets, which minimizes COR.	Allows only a single node to reserve the shared channel.
ARQ-MAC [48]	Allows cooperator nodes to retransmit lost packets, which minimizes E2ED.	A single node can only reserve the channel.
ACP-CA [53]	Introduces an adaptive mechanism to avoid control packet collisions.	Only allows a single transmission at a time, which increases E2ED.
PC-MAC [54]	Avoids collision by using a node’s neighbor’s schedule.	Introduces an additional control packet that increases COR.
CPOR [55]	A node must send one control packet to its neighbors within the same time slot.	Collisions could occur due to hidden terminal and spatio-temporal problems.
IMHEE-MAC [49]	Resolves control packet collisions by randomly assigning a priority number.	Assigning random number may result in excessive delay to transmit critical data.
CFDAMA [50,56]	Seabed nodes use the contention-based approach to reserve the channel, which minimizes E2ED.	Liable to experience THT problems and collisions to control packets may occur.
ED-MAC [30]	Introduces a random mechanism to address THT problems to data packets.	Collisions could occur to beacon packets.
PB-MAC [57]	Relies on a coordinator to schedule data properly.	Introduces an additional control packet, which increases COR.
HTCC [58]	Allows multiple nodes to transmit packets in different directions.	All nodes are forbidden to transmit any packets during the backoff period.
APD-TDMA [59]	Minimizes energy consumption by avoiding collisions of data packets.	Unknown traffic pattern can degrade network performance.

**Table 4:** Summary of the Common Performance Metrics of UWSN MAC Protocols.

Propose/Core Metrics	E2ED	EC	PDR	EE	TC	Thpt	COR	FI
**Contention-free MAC Protocols**								
CBMAC [41]	🗸	🗸	🗸					
GC-MAC [26]		🗸				🗸		🗸
UW-SEEDEX [24]	🗸	🗸	🗸					
**Contention-based MAC Protocols**								
Modified-Slotted-Aloha [37]	🗸	🗸				🗸		
DAP-MAC [52]	🗸				🗸	🗸		
TARS [29]	🗸				🗸	🗸		
RC-SFAMA [44]				🗸		🗸		
MACA-PC [35]		🗸				🗸		
OPMAC [47]	🗸	🗸						
ARQ-MAC [48]				🗸		🗸		
ACP-CA [53]	🗸		🗸			🗸		
PC-MAC [54]	🗸				🗸	🗸		
CPOR [55]	🗸					🗸	🗸	
**Hybrid MAC Protocols**								
IMHEE-MAC [49]		🗸				🗸		
CFDAMA [50,56]	🗸							
ED-MAC [30]			🗸	🗸	🗸	🗸		🗸
PB-MAC [57]	🗸				🗸	🗸		
HTCC [58]						🗸		🗸
APD-TDMA [59]	🗸							

E2ED: End-to-End Delay, EC: Energy Consumption, PDR: Packet Delivery Ratio, EE: Energy Efficiency, TC: Total number of Collisions, Thpt: Throughput, COR: Communication Overhead Ratio, FI: Fairness Index.

**Table 5:** DAMAC Reference Table.

Symbol	Description
Node${}_{i}$	represents the source node i where *i* refers to {1,2,...n}.
B${}_{o}$	represents backoff.
N${}_{oa}$	represents the number of attempts.
M${}_{N_{oa}}$	represents the maximum number of attempts.
T${}_{sn}$	represents the total number of source nodes.
D${}_{p}$	represents the data packets.
Ack${}_{p}$	represents the acknowledgment packet.
C${}_{s}$	represents the carrier sense to check the channel status.
W	represents the period of time a node waits for acknowledgment packet before retransmitting.
R${}_{p}$	represents that the sink node has received the transmitted data packets.
W${}_{r}$	represents an exponential backoff where a node waits a random amount of time to avoid collision.
S${}_{m}$	represents a node to go into sleep mode.
D${}_{ts}$	represents data transmission schedule.

**Table 6:** Simulation Parameters [34] @ 2021 IEEE.

Parameters	Value
Radio propagation model	Underwater Propagation
Channel	UnderwaterChannel
Routing protocol	Vectorbasedforward
Number of nodes	10
Simulation area	1000 m × 125 m
Simulation time	1500 s
Initial energy	10,000 Watts
Transmission power	2.0 Watts
Receiving power	0.75 Watts
Idle power	0.008 Watts
Data rate	10 Kbps
Network load	0.05–0.25 packets/s
Packet size	60 Bytes
Control packet size	5 Bytes
Type of traffic	Constant Bit Rate (CBR)
